# The impact of cigarette smoking on infarct location and in-hospital outcome following acute ST-elevation myocardial infarction

**DOI:** 10.15171/jcvtr.2019.35

**Published:** 2019-08-01

**Authors:** Mehdi Toluey, Samad Ghaffari, Arezou Tajlil, Babak Nasiri, Ali Rostami

**Affiliations:** Cardiovascular Research Center, Tabriz University of Medical Sciences, Tabriz, Iran

**Keywords:** Acute Myocardial Infarction, Tobacco Smoking, In-Hospital Mortality, Inferior Myocardial Infarction

## Abstract

***Introduction:*** Smoking, which is a major modifiable risk factor for coronary artery diseases, affects cardiovascular system with different mechanisms. We designed this study to investigate the association of smoking with location of ST-segment elevation myocardial infarction (STEMI), and short-term outcomes during hospitalization.

***Methods:*** In 1017 consecutive patients with anterior/inferior STEMI, comprehensive demographic, biochemical data, as well as clinical complications and mortality rate, were recorded. Patients were allocated into two groups based on smoking status and compared regarding the location of myocardial infarction, the emergence of clinical complications and in-hospital mortality in univariate and multivariate logistic regression analysis.

***Results:*** Among 1017 patients, 300 patients (29.5%) were smoker and 717 patients (70.5 %) were non-smoker. Smokers were significantly younger and had lower prevalence of diabetes, hyperlipidemia and hypertension. Inferior myocardial infarction was considerably more common in smokers than in non-smokers (45.7% vs. 36%, *P * = 0.001). Heart failure was developed more commonly in non-smokers (33.9% vs. 20%, *P * = 0.001). In-hospital mortality was significantly lower in smokers (6.7% vs. 17.3%, *P * = 0.001). After adjustment for confounding variables, smoking was independently associated with inferior myocardial infarction and lower heart failure [odds ratio: 1.44 (1.06-1.96), *P * = 0.01 and odds ratio: 0.61 (0.40-0.92), *P * = 0.02, respectively]. However, in-hospital mortality was not associated with smoking after adjustment for other factors [odds ratio: 0.69 (0.36-1.31), *P* = 0.2].

***Conclusion:*** Smoking is independently associated with inferior myocardial infarction. Although smokers had lower incidence of heart failure, in-hospital mortality was not different after adjustment for other factors.

## Introduction

Smoking, which is the most lethal modifiable risk factor for coronary artery diseases,^1^ adversely influences cardiovascular system with various mechanisms.^2^ Different policies for decreasing the number of smokers in societies have shown promising result indicating a decline in the incidence of acute coronary syndrome.^3,4^ Despite the established evidence regarding the effects of smoking in increasing premature atherosclerosis,^5,6^ the role of smoking in prognosis of patients after myocardial infarction is not fully recognized.^7-11^ In fact, in some studies, investigating the prognosis of patients after myocardial infarction, surprisingly, smoking was associated with a better prognosis even after considering other influential factors.^9,12^

The fact that smoker patients who are hospitalized for acute coronary syndrome are younger than non-smokers many investigators have contributed the better prognosis of smokers to their younger age and lower prevalence of co-morbidities.^8,11,13,14^ In contrast, some studies reported a favorable prognosis for smokers even after sufficient adjustment for other co-morbidities.^12,15,16^ Though considering all adverse health effects, advice for smoking cession is always the best possible approach.^2,17^ Besides, these controversies may also suggest differences in pathogenesis of coronary diseases in smokers rather than any possible benefits.^8^ Interestingly, in one report published by Alemu et al, smoking had increased the risk of inferior ST-segment elevation myocardial infarction (STEMI) more than anterior STEMI, which may suggest the predilection of harmful effects of tobacco to affect right coronary system more than left coronary system.^18^

So, this study was designed to explore the association of smoking status with the location of myocardial infarction in consecutive patients, hospitalized with anterior or inferior STEMI. In a further analysis, the influence of smoking on the short-term prognosis of patients with STEMI was investigated.

## Materials and Methods

In this retrospective registry-based study, 1017 patients discharged from our hospital, with a final diagnosis of anterior/inferior STEMI during years 2008-2013 were included. Patients were allocated into two group based on smoking status. Comprehensive demographic and serum biochemical data, coronary risk factors including history of hypertension, hyperlipidemia, diabetes, active smoking and family history of premature cardiovascular diseases and history of coronary artery diseases were collected and entered into prepared questionnaires.

First admission electrocardiogram (ECG) in the emergency department was investigated to confirm the registered data regarding the location of myocardial infarction. By evaluating all ECGs of the patients during hospitalization, development of arrhythmia and cardiac bundle branch blocks were determined. Based on the reports of the first transthoracic echocardiographic examination performed during the first day of hospitalization, left ventricular ejection fraction (LVEF), presence of severe mitral regurgitation and any other post-myocardial infarction cardiac abnormalities were documented. Development of heart failure during hospitalization period and in-hospital mortality was recorded for each patient.

All recorded demographic data, risk factors, location of the myocardial infarction, electrocardiographic complications, primary reperfusion therapy and clinical complications including emergence of heart failure and in-hospital mortality was compared between smokers and non-smokers. The patients were also grouped based on the location of myocardial infarction. The incidence of heart failure and in-hospital mortality were compared separately in each group. Independent role of smoking on primary outcomes was investigated by adjustment for other confounding factors.

STEMI was defined as the presence of cardiac chest pain lasting more than 20 minutes with elevated cardiac enzymes and ST elevation of more than 0.15 mV in women or 0.2 mV in men from the J point in leads V2 and V3 or ST elevation of more than 0.1 mV in at least two consecutive leads other than V2/V3. An increase of one point above the 99 percentile cut-off point for MB isoenzyme of creatine kinase (CK-MB) and cardiac troponin I (cTNI) was considered as elevated cardiac enzymes.^19^ Heart failure during the hospitalization period was defined as having pulmonary rales on auscultation and/or signs of congestion on chest X-ray.

### Statistical analysis

Statistical software SPSS (SPSS Inc. Released 2009 PASW Statistics for Windows, Version 18.0) was used to analyze data. Categorical variables were stated as frequencies and percentages. Continuous variables were presented as mean ± standard deviation (SD). Chi-square analysis or Fisher exact test was done as appropriate to compare the frequencies of the categorical variables. To compare continuous variables between two study groups independent *t* test or equivalent non-parametrical Mann-Whitney U-test was used. Multivariate logistic regression analysis was performed to determine the independent role of smoking on location of myocardial infarction, development of heart failure and in-hospital mortality after controlling for other confounding variables. Odds ratios with 95% confidence intervals for developing primary endpoints were stated for smoking status. *P* values less than 0.05 were considered statistically significant.

## Results

Among 1017 patients, 300 patients (29.5%) were smoker and 717 patients (70.5 %) were non-smoker. Inferior STEMI was present in 395 patients (38.83%), and anterior STEMI was present in 622 patients (61.16%). Smokers were significantly younger than non-smokers (56.86 ± 12.3 years vs. 63.32 ± 12.7 years, *P* < 0.001). Smokers were significantly more likely to be male (92.0% vs. 62.9%, *P* < 0.001). Smokers had significantly lower prevalence of hyperlipidemia (16.7% vs. 28.2%, *P* < 0.001). The prevalence of hypertension and diabetes were also lower in smokers (32% vs. 55.2%, *P* < 0.001 and 20% vs. 42.8%, *P* = 0.001, respectively). The family history of premature cardiovascular diseases was lower in smokers (4% vs. 7.5%, *P* = 0.03). History of stroke and coronary artery diseases were not different in two groups (Table 1).

**Table 1:** Baseline characteristics of patients according to smoking status

	**Smokers (n=300)**	**Non-Smokers (n=717)**	***P*** ** value**
Age (y)	56.86±12.3	63.32±12.7	<0.001
Male	277 (92.3%)	452 (63%)	
Female	23 (7.7%)	265 (37%)	<0.001
Hypertension	96 (32.0%)	396 (55.2%)	<0.001
Hyperlipidemia	50 (16.7%)	202 (28.2%)	<0.001
Diabetes	61 (20.3%)	307 (42.8%)	<0.001
Family history	12 (4.0%)	54 (7.5%)	0.03
Chronic sleep apnea	3 (1.0%)	11 (1.5%)	0.7
Coronary artery bypass grafting	6 (2.0%)	13 (1.8%)	0.8
Angioplasty	9 (3.0%)	21 (2.9%)	0.9
Cerebrovascular diseases	8 (2.7%)	33 (4.6%)	0.1
Unstable angina	14 (4.7%)	38 (5.3%)	0.7
Myocardial infarction	26 (8.7%)	56 (7.8%)	0.6
Hematocrit (%)	43.82±5.87	42.37±14.07	0.02
Hemoglobin (g/dL)	14.59±1.92	14.11±4.78	0.03
Creatinine (mg/dL)	1.08±.52	1.30±1.15	0.002
Admission blood glucose (mg/dL)	141.53±83.79	185.24±115.86	<0.001
Total cholesterol (mg/dL)	193.54±57.73	190.16±54.04	0.4
Triglyceride (mg/dL)	164.54±144.14	153.48±120.61	0.2
HDL (mg/dL)	36.87±8.95	38.00±9.70	0.1
Creatine phosphokinase (U/L)*	431.50 (174-1135)	391 (149-934)	0.2
CK-MB (ng/mL)*	58 (35-105.)	52 (33-91)	0.2
cTNI (ng/ml)*	2.60 (0.30-10)	3 (0.50-10)	0.9

*Median (25%-75%) inter-quartile.

The mean hemoglobin and hematocrit level were significantly higher in smokers than in non-smokers (14.59 ± 1.92 mg/dL vs. 14.11 ± 4.78 mg/dL, *P* = 0.03 and 43.82 ± 5.87% vs. 42.37 ± 14.07, *P* = 0.02, respectively). Mean creatinine level was significantly lower in smokers than non-smokers (1.08 ± 0.52 mg/dL vs. 1.30 ± 1.15 mg/dL, *P* = 0.002). Admission blood glucose was also lower in smokers (141.53 ± 83.79 mg/dL vs. 185.24 ± 115.86 mg/dL, *P *≤ 0.001). Serum lipid levels and peak cardiac enzyme levels were not significantly different between two groups (Table 1).

Reperfusion therapy including intravenous fibrinolysis and primary percutaneous coronary intervention (PCI) was used more commonly in non-smokers than in smokers (39% vs. 55.4%, *P *= 0.04). Considering only primary PCI, there was no significant difference between two groups (3.3 % in smokers vs. 5.7 % in non-smokers, *P *= 0.1).

### Clinical features

Among 300 patients in smoker group, 137 cases (45.7%) had inferior STEMI and 258 patients (36%) in non-smoker group had inferior STEMI, which was significantly more common in smokers (*P *= 0.001). There was no significant difference in rate of ventricular fibrillation or tachycardia within first 24 hours after presentation or beyond this time window between two groups. The occurrence of new left bundle branch block, right bundle branch block and atrial fibrillation was not significantly different between smokers and non-smokers. Among patients who underwent coronary angiography, the number of involved vessels was not significantly different between two groups. LVEF of 45% or less was detected in 70% of smokers and 70.6% of non-smokers without significant difference. Smokers had significantly lower incidence of heart failure than non-smokers. Heart failure developed in 20% of smokers and 33.9% of non-smokers (*P* = 0.001). In-hospital mortality was significantly lower in smokers (6.7% vs. 17.3%, *P* = 0.001) (Table 2).

**Table 2:** Clinical and angiographic data of patients according to smoking status

	**Smokers (n=300)**	**Non-smokers (n=717)**	***P*** ** value**
Inferior myocardial infarction	137 (45.7%)	258 (36%)	
Anterior myocardial infarction	163 (54.3%)	459 (64%)	0.001
Ventricular fibrillation (first 24 hours)	12 (4.0%)	43 (6.0%)	0.2
Ventricular fibrillation (other days)	10 (3.3%)	40 (5.6%)	0.1
Left bundle branch block	7 (2.3%)	31 (4.3%)	0.1
Right bundle branch block	14 (4.7%)	40 (5.6%)	0.6
Atrial fibrillation	6 (2.0%)	21 (2.9%)	0.5
Paroxysmal SVT	0 (.0%)	1 (0.1%)	-
First degree atrioventricular block	0 (.0%)	3 (0.4%)	-
Second degree atrioventricular block	1 (0.3%)	2 (0.3%)	1
Third degree atrioventricular block	12 (4.0%)	19 (2.6%)	0.2
Ventricular septal defect	4 (1.3%)	9 (1.3%)	0.9
Mitral regurgitation	91 (30.3%)	278 (38.8%)	0.01
Pulmonary edema	56 (18.7%)	222 (31.0%)	<0.001
Heart failure	60 (20.0%)	243 (33.9%)	<0.001
Ejection fraction ≤45%	210 (70.0%)	506 (70.6%)	0.2
Three vessel disease	47 (15.7%)	124 (17.3%)	0.5
In-hospital mortality	20 (6.7%)	124 (17.3%)	<0.001
Reperfusion therapy	117 (39%)	397 (55.4%)	0.04
Primary-percutaneous coronary intervention	10 (3.3%)	41 (5.7%)	0.1
Angioplasty during hospitalization	56 (18.7%)	146 (20.4%)	0.5

In patients with anterior STEMI, heart failure and in-hospital mortality were higher in non-smokers (35.3% vs. 22.7%, *P* = 0.003 and 19.4% vs. 8%, *P* = 0.001, respectively). In patients with inferior STEMI, heart failure and in-hospital mortality was higher in non-smokers as well (31.4% vs. 16.8%, *P* = 0.002 and 13.6% vs. 5.1%, *P* = 0.01, respectively) (Table 3).

**Table 3:** In-hospital clinical data of patients according to site of STEMI

	**Anterior myocardial infarction n=622**		***P*** ** value**	**Inferior myocardial infarction n=395**		***P*** ** value**
	**Non-Smoker** **n=459**	**Smoker** **n=163**		**Non-Smoker** **n=258**	**Smoker** **n=137**	
Heart failure	162 (35.3%)	37 (22.7%)	0.003	81 (31.4%)	23 (16.8%)	0.002
In-hospital mortality	89 (19.4%)	13 (8.0%)	0.001	35 (13.6%)	7 (5.1%)	0.01

### Comparison of location of myocardial infarction, heart failure and in-hospital mortality with adjustment for other variables

Independent role of smoking on heart failure and in-hospital mortality was investigated in multivariate model. After adjustment for age, gender, hyperlipidemia, hypertension, diabetes, family history, reperfusion therapy, history of ischemic heart diseases, location of myocardial infarction, three-vessel-disease, serum creatinine and serum peak cTNI, smoking, was still independently associated with lower heart failure [odds ratio: 0.61 (0.40-0.92), *P* = 0.02]. However, in-hospital mortality benefit was not associated with smoking after adjustment for other factors [odds ratio: 0.69 (0.36-1.31), *P* = 0.2].

To investigate the relationship of smoking status with development of inferior myocardial infarction, multivariate analysis was performed by adjustment for age, gender, hyperlipidemia, hypertension, diabetes and family history of cardiovascular diseases. Smoking status was independently related to higher inferior myocardial infarction in multivariate analysis [odds ratio: 1.44 (1.06-1.96), *P* = 0.01] (Table 4).

**Table 4:** Effect of Smoking on primary outcomes of the study after controlling for other variables

	**Univariate** **Odds ratio**	**Multivariate** **Odds ratio**	***P*** ** value**
Inferior myocardial infarction^a^	1.49 (1.13-1.96)	1.44 (1.06-1.96)	0.01
Heart failure^b^	0.45 (0.38-0.67)	0.61 (0.40-0.92)	0.02
In-hospital mortality^b^	0.34 (0.21-0.56)	0.69 (0.36-1.31)	0.2

^a^Adjusted for age, sex, hyperlipidemia, hypertension, diabetes, family history. ^b^Adjusted for all above variables and reperfusion therapy, history of ischemic diseases, location of myocardial infarction, three-vessel-disease, serum creatinine, serum cTNI.

## Discussion

According to the results of our study, smoker patients with STEMI are more likely to experience inferior myocardial infarction than anterior myocardial infarction. Although smokers have lower crude mortality rate, the observed difference disappears with adjustments for baseline characteristics including location of myocardial infarction.

Tobacco smoking has various effects on cardiovascular system, which predispose smokers to experience coronary artery disease at a younger age than non-smokers.^2,8^ Although the evidence regarding the effects of smoking on post myocardial infarction survival is conflicting,^7-11^ most recent studies revealed similar prognosis in smokers after myocardial infarction despite their younger age.^7,20,21^ Younger age of patients and as a result low-risk baseline characteristics are the main factors that explain the lower crude mortality rate in smokers when compared to non-smokers.^8^ This is also evident in our study in which consecutive patients with STEMI were included. It is also suggested that smoker patients with STEMI had higher rate of sudden cardiac death before hospital arrival. As a result admitted smoker patients with STEMI may represent low-risk patients with subsequent lower mortality rate.^22^ However, pre-hospital mortality data was not available in our community.

The fact that smoker patients are younger than non-smokers signifies the importance of harmful effects of smoking on cardiovascular health and the potential benefits of smoking cessation as an effective preventive method.^3,20^ Tobacco smoking leads to increased heart rate and blood pressure via the activation of sympathetic nervous system.^23^ Increased oxygen demand occurs simultaneously with vasoconstriction that leads to decreased oxygen supply.^24^ Also, tobacco smoking increases oxidation of LDL cholesterol^25^ and interferes with endothelial function.^26^ Increase in inflammatory factors and acceleration of atherogenesis in combination with increased platelet aggregation and hypercoagulable state contribute to pathogenesis of coronary disease in smokers.^27^ However, these effects may also indicate that there are possible differences in mechanisms of developing myocardial infarction in smokers.

In some studies, therapeutic response after fibrinolysis was greater in smokers.^10,28^ The observed effect is contributed to the higher levels of serum fibrinogen in smokers, which leads to increased fibrin content of thrombosis in smokers.^29^ Increased serum fibrinogen, platelet activity and red blood cell mass also suggest a hypercoagulable state in these patients.^13,27^ The hypercoagulable state may promote coronary thrombosis in smokers.^13,30^ In TEAM-2 study and some other reports, smokers had greater thrombus burden than plaque burden. Also, they were more likely to have TIMI grade flow 3 after thrombolytic therapy.^7,29^ These findings support the dominance of thrombogenic mechanism in pathogenesis of STEMI in smoking patients.^27,31,32^

Our findings regarding the effects of smoking on location of myocardial infarction, is consistent with the results of a study by Alemu et al. They investigated the association of smoking status with the location of myocardial infarction in a pooled data from five different cohorts as well as their cohort. Both analyses revealed higher risk of inferior myocardial infarction in smoker patients.^18^ In another study by Grines et al in which they have studied the role of smoking status on mortality of patients, the prevalence of inferior STEMI was 60% in smokers and 53% in non-smokers, which was significantly higher in smoker group. Although the overall prevalence of inferior STEMI was higher in their study, the results regarding the association of smoking with inferior STEMI were similar to our finding.^30^

As shown in our study, smokers have higher rate of inferior STEMI than anterior STEMI. The dominant thrombogenic etiology of STEMI in smokers may explain the higher rate of inferior STEMI, in which right coronary artery (RCA) is the infarct-related artery in majority of cases.^33^ RCA is a less branching coronary artery in comparison to left coronary artery, and this may facilitate formation of large clots in this vessel. Also, RCA has less turbulent flow that in conjunction with its larger diameter may predispose it to thrombus formation.^34^ However, the exact underlying mechanism by which smoking increases the risk of inferior STEMI needs further investigations.

The role of smoking in distribution of coronary lesions has been investigated in different studies with conflicting results.^35-39^ According to a paper published by Zwaag et al, smoking increases the risk of RCA lesions more than other vessels.^39^ Koliaki et al reported a positive correlation between smoking and presence of a lesion in RCA, left circumflex artery and left anterior descending artery (LAD) but not left main coronary artery (LMCA).^36^ In a study by Castela et al, smokers had lower prevalence of LMCA and proximal LAD lesions, but this finding was not statistically significant.^38^ Another study Aygul et al have shown no association between smoking and infarct-related artery in men and women.^37^ In another report, Köz et al investigated the effect of coronary risk factors on lesion distribution in LMCA and proximal or mid-LAD in comparison to other lesions and found no association between the lesions and smoking status.^35^

### Limitations of study

In this study, we did not separate current smokers from former smokers. In addition, we did not determine the accurate number of smoked cigarettes per day or length of smoking history for each patient. Various brands of cigarettes may contain different substances; however, we did not have the information regarding the cigarette brands used by patients. It should be mentioned that we only included patients who presented to our hospital and out-of-hospital deaths due to STEMI were not available. In our survey, which was a single-center observational study, use of reperfusion therapies including thrombolytic therapy and PCI was lower than current standards.

## Conclusion

Smoking increases the risk of inferior STEMI even after adjustment for other confounding factors. The rate of development of heart failure after STEMI is lower in smokers. However, in-hospital mortality rate in smokers is not different in smokers and no-smokers after adjustment for confounding factors.

## Competing interests

Authors declare no conflict of interests.

## Funding

None.

## Ethical Approval

The study protocol was reviewed and accepted by the Institutional Review Board Committee at our University of Medical Sciences. It was exempted from obtaining informed consent due to its descriptive design. However, complete patient confidentiality was preserved during the whole study.
